# Opening a Band Gap in Biphenylene Monolayer via Strain: A First-Principles Study

**DOI:** 10.3390/molecules28104178

**Published:** 2023-05-18

**Authors:** Yinlong Hou, Kai Ren, Yu Wei, Dan Yang, Zhen Cui, Ke Wang

**Affiliations:** 1 School of Automation, Xi’an University of Posts & Telecommunications, Xi’an 710121, Chinayangdan1990@xupt.edu.cn (D.Y.); 2 School of Mechanical and Electronic Engineering, Nanjing Forestry University, Nanjing 210042, China; 3 School of Automation and Information Engineering, Xi’an University of Technology, Xi’an 710048, China

**Keywords:** 2D biphenylene, electronic properties, indirect band gap, single phonon mode

## Abstract

A biphenylene network is a novel 2D allotropy of carbon with periodic 4-6-8 rings, which was synthesized successfully in 2021. In recent years, although the mechanical properties and thermal transport received a lot of research attention, how to open the Dirac cone in the band structure of a biphenylene network is still a confused question. In this work, we utilized uniaxial and biaxial lattice strains to manipulate the electronic properties and phonon frequencies of biphenylene, and we found an indirect band gap under 10% biaxial strain through the first-principles calculations. This indirect band gap is caused by the competition between the band-edge state A and the Dirac cone for the conduction band minimum (CBM). Additionally, the lightest carrier’s effective mass in biphenylene is 0.184 *m*_0_ for electrons along x (Γ→X) direction, while the effective mass for holes shows a remarkable anisotropy, suggesting the holes in the tensile biphenylene monolayer are confined within a one-dimensional chain along x direction. For phonon dispersion, we discovered that the Raman-active $A_{g}^{3}$ phonon mode shows a robust single phonon mode character under both compressive and tensile strain, but its frequency is sensitive to lattice strain, suggesting the lattice strain in biphenylene can be identified by Raman spectroscopy

## 1. Introduction

The development of materials is critical to design and fabricate novel sensors, actuators, and resonators, which are fundamental to the engineering systems of the future. Nowadays, engineering products can be innovative only if they ensure safety, reliability and good quality of life in all parts of the world, requiring the new materials to be green and smart as much as possible [1]. As known, carbon is one of the most widely distributed elements in nature, whose allotropes are green and suitable for innovative engineering products in the future. In 2004, graphene, a two-dimensional (2D) allotrope of carbon, was fabricated by mechanical stripping [2]; then, the high carrier mobility [3], superb room-temperature thermal conductivity [4,5], and excellent fracture strength [6] in graphene encouraged substantial experimental and theoretical researchers to explore 2D materials [7,8,9]. In the past two decades, the number of members in the 2D family increased rapidly, and numerous 2D allotropes of carbon were included, such as metallic R-graphene [10], commensurate graphene [11], ψ-graphyne [12], Stone–Wales graphene [13], topological phagraphene [14], penta-graphene [15], and twin graphene [16]. In 2021, Fan et al. [17] synthesized a biphenylene network with periodic 4-6-8 carbon rings using an on-surface interpolymer dehydrofluorination reaction. Subsequently, the electronic [18,19,20], mechanical [21,22,23], and thermal [24,25] properties of a biphenylene network were investigated deeply by the first-principles calculations. In 2021, a pair of type-Ⅱ Dirac cones in the band structure of a biphenylene network was predicted by first-principles calculations [26], while Zhang et al. [27] obtained orientation-dependent lattice thermal conductivities of biphenylene with and without hydrogenation by solving the phonon Boltzmann transport equation (BTE). Recently, Ren et al. [28] tuned the mechanical anisotropy of the biphenylene monolayer from 1.2 to 1.33 by N doping, while Ren et al. [29] employed the first-principles calculations to investigate the effect of atom doping on the magnetism in the biphenylene monolayer, and found Fe, Cl, and Cr doping can induce magnetic moment into biphenylene. However, how to open a band gap in biphenylene remains to be solved, as the metallic behavior of biphenylene strongly hinders its application in photodetectors [30,31] and photocatalysis [32,33].

Strain is an effective and frequently used method to manipulate the electronic [34,35], catalytic [36,37,38,39], thermal [40,41], and magnetic [42,43] properties of 2D materials. For instance, Gui et al. [44] found that the pseudogap of graphene decreased linearly with the strain strength in the elastic regime through first-principles calculations, while Wang et al. [45] reported the strain tailoring for the magnetic phase transition of the layered antiferromagnetic semiconductor CrSBr. In practice, strain can be induced into 2D materials either intentionally or naturally. During the preparation and transfer of 2D materials, strain can be introduced naturally, due to a higher or lower lattice mismatch between the crystal and substrate. The uniaxial strain can be also induced intentionally by bending the substrates where 2D materials are elongated without slippage, while biaxial strain can be resulted by surface corrugations or lattice mismatch [46,47]. For biphenylene, Feng et al. [48] unveiled that biaxial compressive strains could improve the catalytic performance, while Yang et al. [49] addressed an unexpected strain-enhanced thermal conductivity of the biphenylene monolayer by solving phonon BTE with the machine learning interatomic potential. However, the influences of strain on the electronic properties of biphenylene still need exploration.

In this work, we investigated the influences of strain on the electronic and phononic properties of biphenylene through the first-principles calculations and found an indirect band gap of 0.07 eV in biphenylene when 10% biaxial strain was applied, because of the competition between the band-edge state A and Dirac cone for the conduction band minimum (CBM). Subsequently, the carrier’s effective mass, work function and phonon frequency under strain were also explored, revealing the carrier’s mobility, contact resistance, and Raman spectroscopy of biphenylene can be manipulated effectively by lattice strain. In addition, a single phonon mode $A_{g}^{3}$ with frequency of up to 49.67 THz at Γ point has also been found in biphenylene, rendering the biphenylene a promising candidate in novel quantum nonlinear elements, phonon lasers, and quantum mechanical resonators. More interestingly, the resonant frequency of this quantum mechanical resonator based on biphenylene can be controlled by strain effectively, due to the large Grüneisen constant of the single phonon mode $A_{g}^{3}$. These results are beneficial for the application of biphenylene in next-generation novel electronic and mechanical devices.

## 2. Results and Discussion

### 2.1. Geometrical Structure and Electronic Properties of Original Biphenylene

**Figure 1:**
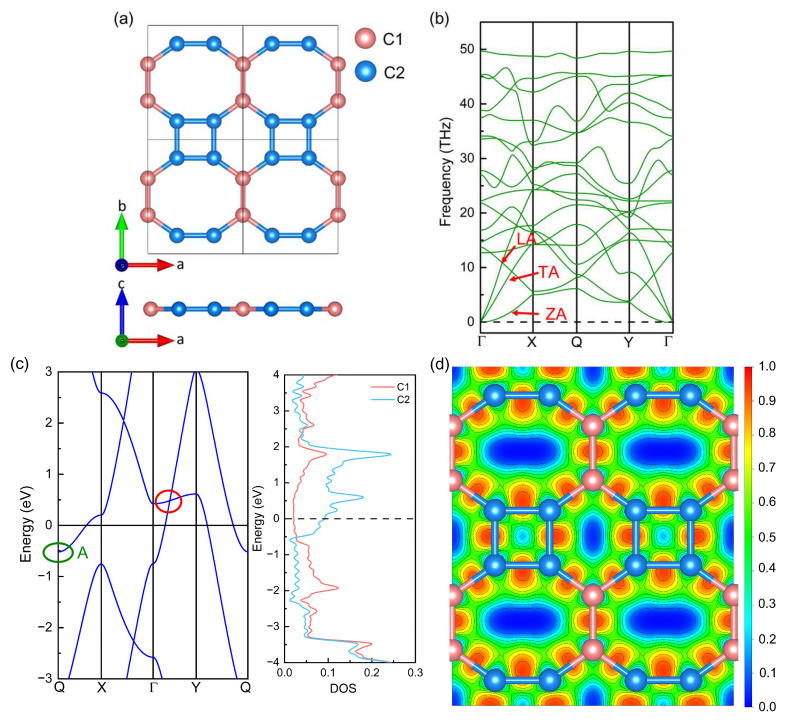
Geometrical structure (**a**), phonon dispersion (**b**), band structure and partial density of states (pDOS) (**c**), and electron localization function (ELF) (**d**) of biphenylene monolayer. In Figure 1b, red arrows mark the three acoustic phonon modes (LA, TA, and ZA); the red and green circles in Figure 1c mark the Dirac cone and band-edge state A, respectively.

Before applying strain, we relaxed firstly the original biphenylene monolayer, and calculated its electronic properties and phonon dispersion to use as the benchmark. The top and side views of the biphenylene network are presented in Figure 1a, where four-, six- and eight-membered rings are formed by sp^2^-hybridized C atoms. Based on this periodic 4-6-8 ring, the biphenylene network belongs to an orthorhombic lattice with space group of Pmmm (No. 47). The lattice constants of the biphenylene monolayer are *a* = 3.761 Å and *b* = 4.519 Å after structural relaxation, which agrees well with previous experimental and theoretical results [17,25,27]. In the unit cell of biphenylene, there are six C atoms, which can be divided into two types (C1 and C2) and represented by pink and blue balls in Figure 1a, respectively. The lengths of C1-C1, C1-C2, and C2-C2 bonds are 1.448 Å, 1.407 Å, and 1.456 Å, respectively, while the C2-C2-C2, C2-C2-C1, and C1-C1-C2 bond angles are 90°, 144.97°, and 125.03° in the biphenylene monolayer. To identify the dynamical stability of our relaxed biphenylene network, the phonon dispersion was calculated and exhibited in Figure 1b. In phonon dispersion, there are 18 phonon branches, including 15 optical and 3 acoustic phonon modes, which is consistent with the number of C atoms in the unit cell of the biphenylene monolayer. These acoustic phonon modes are named longitudinal acoustic (LA), transverse acoustic (TA), and out-of-plane acoustic (ZA) modes, as shown in Figure 1b. For ZA acoustic phonon modes, there are some ultra-small imaginary frequencies at the center of the Brillouin zone, indicating the biphenylene monolayer is unstable to the long-wavelength periodic distortions. A similar phenomenon has also been found in graphene [50] and CrI_3_ [51] monolayers.

Based on the relaxed biphenylene monolayer, we calculated the band structure, partial density of state (pDOS), and electron localization function (ELF), as shown in Figure 1c,d where the Fermi level was set as 0 eV. In Figure 1c, the Dirac cone (marked by the red circle in Figure 1c) near the Fermi level can be found in the Γ → Y path, consistent with the results of Bafekry [52] and Luo [23]. Furthermore, the biphenylene monolayer shows a metallic behavior, because both the conduction band minimum (CBM) and valence band maximum (VBM) go across the Fermi level. The CBM is represented by band-edge state A (marked by the green ellipse in Figure 1c) and its value is −0.517 eV, while the VBM is dominated by the Dirac cone and its value is 0.481 eV. This metallic behavior of the biphenylene monolayer can also be identified by the pDOS. In the pDOS, the pink and blue curves plot the contribution of every C1 and C2 atom to the DOS, respectively. Obviously, the DOS of C2 atoms around the Dirac cone and band-edge state A is much higher than that of C1 atoms, suggesting the domination of C2 atoms to the Dirac cone and band-edge state A. To understand the underlying characteristics of bonding properties in the biphenylene monolayer, the electron localization function (ELF) from the aspect of (0 0 −1) plane was quantified and illustrated in Figure 1d. The value of ELF always is between 0 and 1, which can be obtained through the VASP calculation [53] and the VASPKIT program [54]. If the value of ELF is below 0.5, the bonding would be regarded as ionic. If this value is in the range of 0.5~1.0, covalent bonding would be suggested. In Figure 1d, the largest value of ELF between C atoms in the biphenylene monolayer is ~0.948, much larger than 0.5, indicating strong covalent bonding.

### 2.2. Effect of Strain on Electronic Properties and Geometrical Structure

Strain within the range of ±10% was imposed to the biphenylene monolayer by stretching or compressing the lattice of the relaxed biphenylene monolayer. The definition of strain is $\varepsilon =\frac{a_{x(y)}{-a}_{x0(y0)}}{a_{x0(y0)}}\times 100\%$, where $a_{x\left(y\right)}$ and $a_{x0\left(y0\right)}$ are the lattice constants of strained and original biphenylene monolayers along the *x* (*y*) axis, respectively. A positive value of ε corresponds to lattice expansion, whereas a negative value refers to compression. Under each applied strain, the atom positions were fully relaxed through the technique of energy minimization to ensure the Hellmann–Feynman force on atoms reached the convergence limit (10^−4^ eV/Å). The bond lengths and angles of the strained biphenylene monolayer are shown in Table 1 and Table 2. In Table 1, we can find that biaxial strain has the largest influence on all three bond lengths. For instance, −10% biaxial train compresses the C1-C1, C1-C2, and C2-C2 bond lengths by 0.176 Å, 0.141 Å, and 0.153 Å, respectively, while the −10% *x*-axial strain only decreases these three bond lengths by 0.076 Å, 0.073 Å, and 0.116 Å. Additionally, it can also be discovered that the *x*-axial strain has more impressive impact on the C2-C2 bond length than the *y*-axial strain, whereas the length of the C1-C1 bond is more affected by the *y*-axial strain, due to the orientation of C1-C1 and C2-C2 bonds in Figure 1a. Moreover, the bond angles between C1 and C2 atoms increase with lattice compression but reduce with lattice expansion, while the C2-C2-C2 bond angle keeps as 90° and is robust to the uniaxial and biaxial strains. Furthermore, both uniaxial and biaxial strains within the range of ±10% do not destroy the periodic 4-6-8 rings in the biphenylene monolayer.

**Figure 2:**
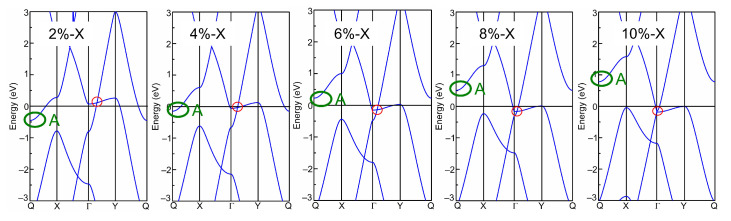

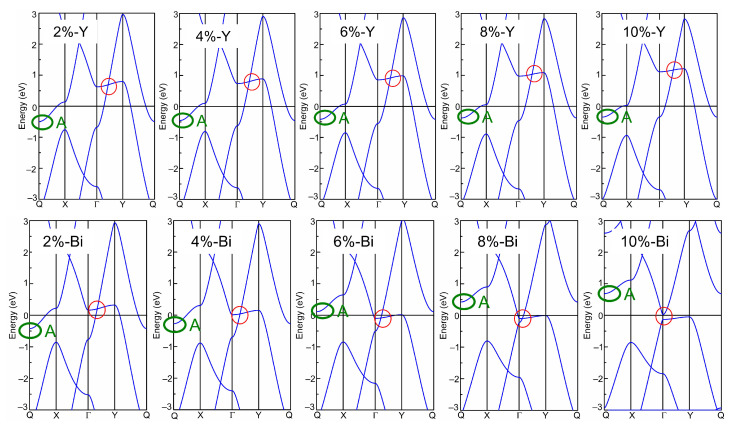
Band structure of biphenylene monolayer under uniaxial (along *x* or *y* direction) and biaxial (along both *x* and *y* direction, Bi) lattice expansion. The red and green circles mark the Dirac cone and band-edge state A, respectively.

**Figure 3:**
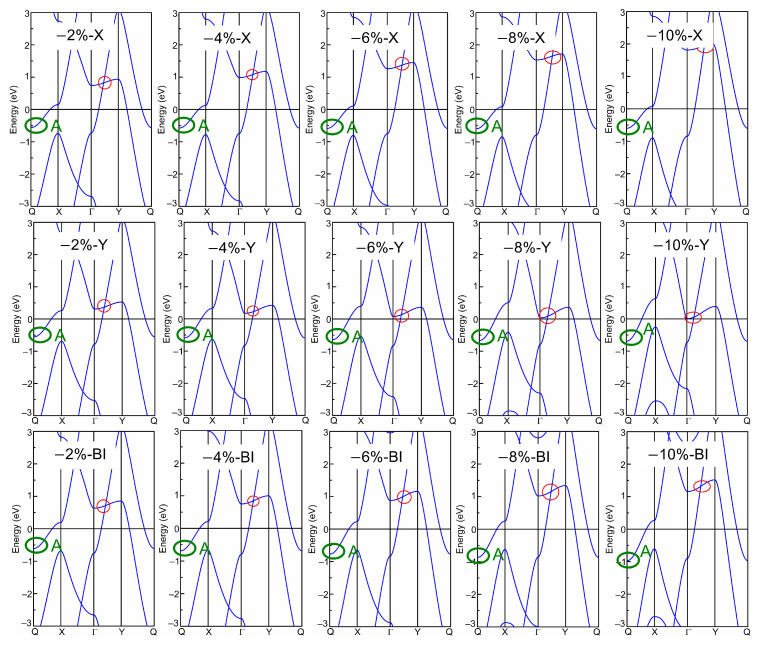
Band structure of biphenylene monolayer under uniaxial (along *x* or *y* direction) and biaxial (Bi) compressions. The red and green circles mark the Dirac cone and band-edge state A, respectively.

Afterwards, the band structures of the strained biphenylene monolayer were calculated using the PBE exchange–correlation functional. The calculated band structures of the biphenylene monolayer with uniaxial (along *x* or *y* direction) and biaxial (along both *x* and *y* direction) expansion are presented in Figure 2, while that of the compressive biphenylene monolayer are given in Figure 3. In Figure 3, the metallic state is unchanged by compressive uniaxial and biaxial strains within the range of −10%~−2%, whereas the Dirac cone is opened when the biaxial tensile strain reaches 10%, resulting in a semiconducting state with an indirect band gap of 0.07 eV. In this case, the carriers’ effective masses were calculated as 0.184 *m*_0_ (for electrons) and 0.229 *m*_0_ (for holes) along *x* (Γ → X) direction by $\hbar^{2}{\left[\partial^{2}E\left(k\right)/\partial k_{\alpha }\partial k_{\beta }\right]}^{-1}$ [55], while these are 0.203 *m*_0_ (for electrons) and 7.705 *m*_0_ (for holes) along *y* (Γ → Y) axis. Among these values, the lightest effective mass is 0.184 *m*_0_ for electrons along *x* direction, slightly heavier than that in black phosphorene (0.17 *m*_0_) but much lighter than that in MoS_2_ monolayer (0.47 *m*_0_) [56,57]. As known, the carrier effective mass is strongly related to the mobility, and a heavy carrier effective mass leads to a low mobility. Therefore, it can be concluded that electrons own the highest mobility along *x*-axis in the tensile biphenylene monolayer with biaxial strain of 10%. Additionally, the anisotropy of mobility is up to ~33.65 for holes, indicating the holes in the tensile biphenylene monolayer are confined within one-dimensional chain along the *x* direction.

**Figure 4:**
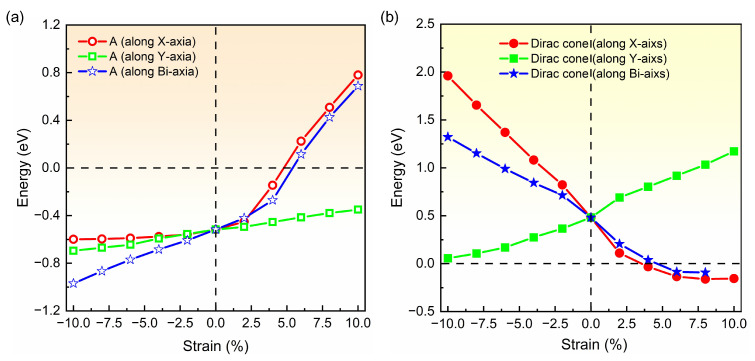
Energies of band-edge state A (**a**) and Dirac cone (**b**) in the band structure of biphenylene monolayer as functions of applied strain. The red, green and blue lines represent the function of strain applied in the *x*-, *y*- and bi-axial.

Based on band structure in Figure 2 and Figure 3, it can be seen that the semiconducting state with an indirect band gap in the strained biphenylene monolayer is induced by the competition between the band-edge state A and the Dirac cone for the CBM. To observe the shifts of the band-edge state A and Dirac cone explicitly, we extracted and plotted the energies of these states versus the strain in Figure 4, where the Fermi level is also set as 0 eV. In Figure 4a, it can be found that band-edge state A is more sensitive to lattice expansion than compression. For instance, the largest variation of band-edge state A with compression is 0.45 eV, much smaller than that with expansion (1.23 eV). When biaxial strain is applied, the energy of band-edge state A elevates from −0.97 eV to 0.69 eV and crosses the Fermi level. A similar change can also be observed when strain is applied in *x* direction. For the Dirac cone, lattice compression elevates its energy, while lattice expansion reduces its energy. As the *y*-axial strain increases from −10% to 10%, the energy of the Dirac cone blueshifts from 0.06 eV to 1.17 eV, and remains above the Fermi level. When strain is applied in *x* axial and changes from −10% to 10%, the energy of the Dirac cone decreases from 1.96 eV to −0.16 eV and moves to the Γ point, which is similar to the phenomenon under biaxial strain within the range of −10%~8%. In addition, it is suggested that the *x*-axial strain has more impressive impacts on the Dirac cone than *y*-axial strain. This is because C2 atoms arranged along *x* axial dominate the DOS at the Dirac point, as shown in Figure 1c. Furthermore, when the *x*- or bi-axial strain exceeds 6%, the energy of the Dirac cone is lower than the band-edge state A, thus representing the CBM. Once the biaxial strain reaches 10%, the Dirac cone near Fermi level is opened, and an indirect band gap between the CBM at Γ point and the VBM at Y point is induced in the strained biphenylene monolayer.

**Figure 5:**
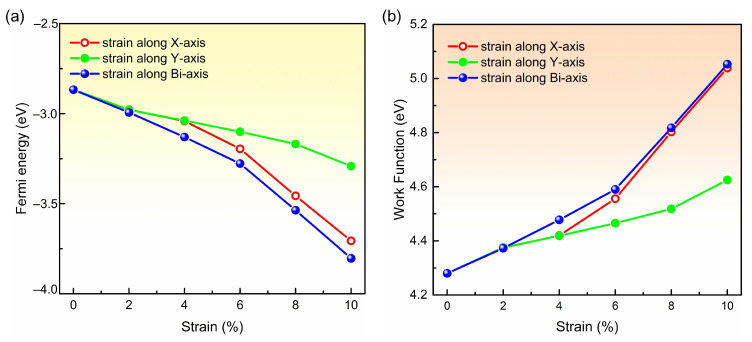
Variation of the Fermi energy (**a**) and work function (**b**) for biphenylene monolayer with tensile strain. The red, green and blue lines represent the variation with strain applied in *x*-, *y*- and bi-axial.

In addition, the work functions were calculated by subtracting the Fermi energy from the electrostatic potential in the middle of vacuum [58,59]. We plot the Fermi energies and work functions of biphenylene monolayers with and without tensile strain in Figure 5a,b, respectively. It is apparent that the Fermi energy decreases with strain increasing, regardless of the direction in which the tensile strain is applied. In more detail, the Fermi energy reduces slowly from 0% (−2.87 eV) to 6% (−3.19 eV) as strain is applied in *x* direction, and then decreases rapidly from 6% (−3.19 eV) to 10% (−3.71 eV). When the tensile strain is applied in *y* direction, the Fermi energy shows a flat downtrend from 0% (−2.87 eV) to 10% (−3.29 eV). Differently, biaxial strain leads to a steeper descent from 0% (−2.87 eV) to 10% (−3.80 eV). Correspondingly, the work function in the biphenylene monolayer without any strain is 4.28 eV, smaller than that in graphene (4.5 eV) [60]. Subsequently, the work function grows significantly with tensile strain, and the growths are up to 0.76 eV, 0.35 eV, and 0.77 eV for *x*-, *y*- and bi-axial strains, respectively. These remarkable growths in work function reveal the possibility to tune the contact resistance by controlling the lattice expansion of biphenylene crystal and selecting suitable contact metals.

### 2.3. Effect of Strain on Phonic Properties

**Figure 6:**
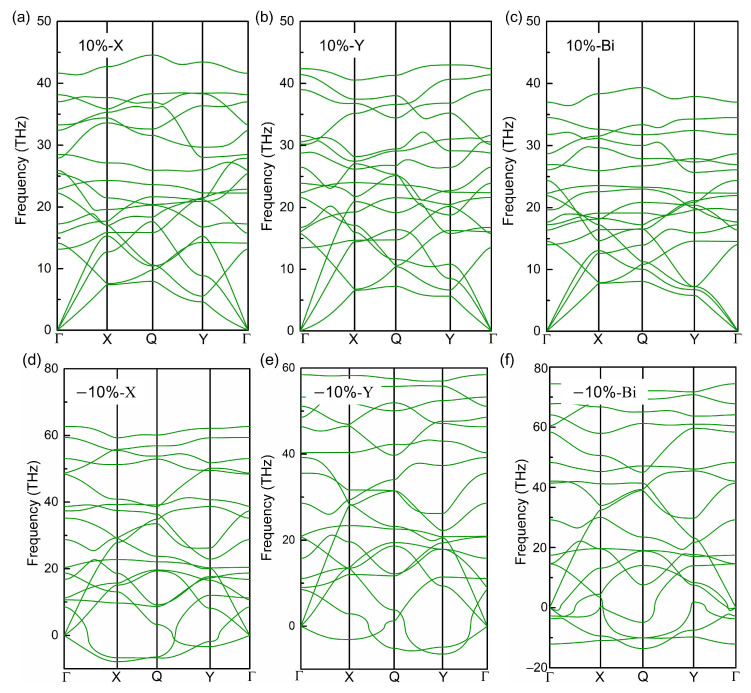
Phonon dispersion of biphenylene as ±10% *x*- (**a**,**d**), *y*- (**b**,**e**) and bi-axial (**c**,**f**) strain is applied.

In 2019, Yin et al. [40] reported the impressive impact of strain and carrier doping on the phononic properties of graphene-like borophene, such as phonon frequency. Here, how does the strain affect the phononic properties of the biphenylene monolayer? To answer this question, we calculated and showed the phonon dispersions of the biphenylene monolayer under ±10% strain in Figure 6. As known, there are 3*n* branches in phonon dispersion for a crystal with *n* atoms per unit cell, including three acoustic (LA, TA, ZA phonons in Figure 1b) and 3*n*-3 optical modes. For three acoustic phonon modes, their frequencies are zero at Γ point in the irreducible Brillouin zone, and their eigenvectors give the translation of C atoms in unit cell along *x*-, *y*- and *z*-directions, respectively. Generally, the dynamical stability of crystal can be identified by the imaginary frequency of three acoustic branches in phonon dispersion. In our study, there are several remarkable imaginary frequencies in phonon dispersion when 10% compressive strain is applied, especially at the boundary of Brillouin zone, revealing the dynamic instability of the biphenylene monolayer under large compressive strain. Wang et al. [61] calculated the Grüneisen constants of phonon modes for the biphenylene monolayer, and found the Grüneisen constants for all three acoustic branches are negative at the boundary of the Brillouin zone, suggesting phonon frequency redshift with lattice compression. Therefore, it is supposed that the imaginary phonon frequencies under 10% compressive strain are strongly related to these negative Grüneisen constants. When the applied strain is tensile, there is no imaginary frequency in phonon dispersion, even when the applied tensile strain is up to 10%. Compared with the slight imaginary frequency of ZA mode in Figure 1b (phonon dispersion of the original biphenylene monolayer), it can be found that 10% tensile strain improves the stability to the long-wavelength periodic distortions, which is desirable for the applications of 2D materials in next-generation electronic devices. However, a problem arises of whether the dynamical stability of the biphenylene monolayer is kept or not, when a bending is applied to substrate. During the bending of the substrates, tensile strain is induced to one surface while compression occurs in the opposite surface of the biphenylene monolayer. In this case, it is difficult to guarantee the dynamical stability of the biphenylene monolayer, because the compression leads to the reduction of phonon frequencies for three acoustic branches at the boundary of the Brillouin zone, but the tensile strain enlarges their frequencies.

In addition to three acoustic phonon modes, there are 15 optical phonon modes in the phonon dispersion of the biphenylene monolayer, whose eigenvectors describe the relative motion of C atoms in the unit cell of the biphenylene monolayer. What is the effect of strain on the optical phonons? Recently, Wang et al. [61] reported a Raman-active single phonon mode ($A_{g}^{3}$) with frequency of 49.67 THz at Γ point in an original biphenylene monolayer, which is rare in other 2D materials, such as MoS_2_ [62], black phosphorene [63] and graphene [25]. Single phonon mode without any degeneracy is promising for the design of mechanical resonators, and a large phonon frequency (*f*) leads to a wide range of cooling temperature ($T\;\leq {\;\mathrm{hf}/k}_{B}$) for a mechanical resonator [64]. In the biphenylene monolayer, the large frequency of Raman-active $A_{g}^{3}$ mode corresponds to the cooling temperature ranging from 0 to ~2366.6 K, which renders the biphenylene monolayer a promising candidate in novel quantum nonlinear elements, phonon lasers, and quantum mechanical resonators. To observe the influence of ±10% strain, we extracted and listed the frequencies of 15 optical phonon modes at Γ point in Table 3. In our work, the Raman-active $A_{g}^{3}$ mode is still a single phonon mode without any degeneracy, and its frequency is elevated to 62.69 THz (*x*), 58.46 THz (*y*), and 74.44 THz (bi) by 10% uniaxial and biaxial lattice compression, respectively, leading to significant widening of the cooling temperature range. When 10% tensile strain is applied, the frequency of Raman-active $A_{g}^{3}$ mode reduces to 41.62 THz (*x*), 42.39 THz (*y*), and 37.0 THz (bi). These noticeable changes in the frequency of Raman-active $A_{g}^{3}$ mode agree with its large Grüneisen constant of 2.07 at Γ point, allowing to control the resonant frequency of this quantum mechanical resonator based on biphenylene by strain and identify the lattice strain of biphenylene through Raman spectroscopy.

## 3. Computational Details

All of the first-principles calculations were implemented by the Vienna ab initio simulation package (VASP) with framework of density functional theory [65,66]. In all calculations, the Perdew–Burke–Ernzerhof (PBE) method of general gradient approximation (GGA) was selected as the exchange–correlation functional [67,68]. Moreover, the convergence limits of energy and the Hellmann–Feynman force were set as 10^−8^ eV and 10^−4^ eV/Å, respectively, when the geometrical structure was optimized using 800 eV cutoff energy and a 6 × 5 × 1 Monkhorst–Pack (MP) grid. A denser MP grid of 11 × 9 × 1 was used in self-consistent calculations for better results of band structure and density of states (DOS). To suppress the non-physical interaction between adjacent layers, a 20 Å vacuum space was imposed along the out-of-plane direction. To calculate phonon dispersion, a 14 × 12 × 1 MP grid was used for a 2 × 2 × 1 supercell, and then phonon frequencies were obtained by PHONOPY code based on density function perturbation theory (DFPT) [69]. 

## 4. Conclusions

In summary, we employed the first-principles calculations to investigate the influences of strain on the electronic properties and phonon frequency of biphenylene. We found an indirect band gap in biphenylene under 10% biaxial lattice expansion, due to the competition between the band-edge state A and the Dirac cone for the CBM. In this case, the lightest effective mass is 0.184 *m*_0_ for electrons along x (Γ → X) direction, resulting in a comparable mobility with black phosphorene. The effective mass for holes shows a remarkable anisotropy, suggesting the holes in the tensile biphenylene monolayer are confined within a one-dimensional chain along *x* direction. Moreoever, the work function increases obviously from 4.28 eV to 5.04 eV, 4.63 eV, and 5.05 eV with *x*-, *y*-, and bi-axial strain, respectively, which allows the modification of contact resistance between biphenylene and electrode by lattice strain. Additionally, the strain also has an impressive impact on phonon dispersion. Compressive strain with amplitude of 10% leads to several imaginary frequencies in phonon spectrum and destroys the dynamical stability of biphenylene. The Raman-active phonon mode $A_{g}^{3}$ shows a robust single phonon mode character under 10% compressive and tensile strain. Furthermore, the frequency of Raman-active $A_{g}^{3}$ mode behaves significantly different under 10% compressive and tensile strains, revealing that Raman spectroscopy can be used to detect the lattice deformation of biphenylene. These findings offer an insightful view to understand the strain tailoring of biphenylene, and we hope it could promote the study and application of novel carbon allotropes.

## Figures and Tables

**Table 1:** Lengths for C1-C1, C1-C2, and C2-C2 bonds in the strained biphenylene monolayer.

Strain (%)	C1-C1 Bond Length (Å)			C1-C2 Bond Length (Å)			C2-C2 Bond Length (Å)		
	*x*	*y*	bi	*x*	*y*	bi	*x*	*y*	bi
−10	1.372	1.344	1.272	1.334	1.347	1.266	1.340	1.424	1.303
−8	1.388	1.361	1.305	1.348	1.359	1.295	1.360	1.433	1.333
−6	1.403	1.380	1.338	1.362	1.371	1.323	1.382	1.441	1.363
−4	1.419	1.400	1.373	1.377	1.383	1.352	1.405	1.448	1.393
−2	1.433	1.423	1.408	1.433	1.395	1.380	1.428	1.452	1.422
0	1.448	1.448	1.448	1.407	1.407	1.407	1.456	1.456	1.456
2	1.462	1.473	1.491	1.422	1.420	1.433	1.491	1.456	1.496
4	1.475	1.503	1.537	1.436	1.432	1.459	1.530	1.460	1.534
6	1.489	1.536	1.593	1.447	1.443	1.480	1.576	1.464	1.588
8	1.498	1.575	1.664	1.462	1.453	1.497	1.612	1.466	1.630
10	1.504	1.620	1.751	1.476	1.461	1.510	1.649	1.466	1.673

**Table 2:** Bond angles between C1-C1-C2, C2-C2-C1, and C2-C2-C2 in biphenylene monolayer with strain.

Strain (%)	C1-C1-C2 Bond Angle (°)			C2-C2-C1 Bond Angle (°)			C2-C2-C2 Bond Angle (°)		
	*x*	*y*	bi	*x*	*y*	bi	*x*	*y*	bi
−10	129.94	119.81	124.69	140.06	150.19	145.31	90	90	90
−8	128.82	121.03	124.75	141.18	148.97	145.26	90	90	90
−6	127.75	122.17	124.82	142.24	147.83	145.18	90	90	90
−4	126.76	123.22	124.85	143.24	146.78	145.15	90	90	90
−2	125.80	124.14	124.87	144.20	145.86	145.13	90	90	90
0	125.03	125.03	125.03	144.97	144.97	144.97	90	90	90
2	124.42	125.74	125.27	145.58	144.26	144.73	90	90	90
4	123.97	126.52	125.41	146.03	143.48	144.59	90	90	90
6	123.61	127.22	125.84	146.39	142.78	144.16	90	90	90
8	123.05	127.83	125.70	146.95	142.17	144.30	90	90	90
10	122.56	128.27	125.33	147.44	141.73	144.67	90	90	90

**Table 3:** Frequencies of 15 optical modes in the phonon spectrum of unstrained and strained biphenylene monolayers. The phonon frequency of unstrained biphenylene monolayer is from Ref. [61].

Phonon Mode	Phonon Frequency *f* (THz)						
	−10%*-x*	−10%*-y*	−10%-bi	Unstrained	10%-*x*	10%-*y*	10%-bi
$B_{3g}^{1}$	8.55	8.52	−2.75	12.70	13.19	13.51	14.02
$B_{1u}^{\;}$	10.68	9.01	−12.09	13.73	14.16	15.81	14.57
$B_{2g}^{\;}$	11.27	11.08	−3.66	14.69	15.76	16.11	16.38
$B_{1g}^{1}$	16.82	15.79	14.64	16.68	17.28	16.80	17.25
$B_{3g}^{2}$	18.71	20.74	14.55	21.86	22.25	21.61	17.70
$A_{u}^{\;}$	20.43	20.91	17.44	22.20	22.88	22.39	19.65
$A_{g}^{1}$	28.75	28.59	29.18	27.03	25.42	23.90	21.95
$B_{1g}^{2}$	35.18	35.54	41.40	27.80	25.91	26.51	22.32
$B_{2u}^{1}$	37.37	39.21	42.07	33.58	27.89	28.79	24.40
$B_{3u}^{1}$	38.72	40.32	48.28	34.11	28.48	30.19	26.10
$B_{3u}^{2}$	48.42	46.37	58.39	37.46	32.40	30.60	26.93
$A_{g}^{2}$	48.71	48.56	60.51	38.84	33.28	31.61	28.70
$B_{1g}^{3}$	53.06	51.11	64.10	45.18	37.02	39.02	31.79
$B_{2u}^{2}$	59.40	53.26	67.81	45.24	38.15	41.41	34.52
$\left(A_{g}^{3}\right)$	62.69	58.46	74.44	49.67	41.62	42.39	37.00

## Data Availability

The data presented in this study are available upon request from the corresponding author.

## Sample Availability

Samples of the compounds are available from the authors.
